# Target-specific cardiotoxicity of tyrosine kinase inhibitors: a Systematic Review and meta-analysis

**DOI:** 10.3389/fphar.2026.1807099

**Published:** 2026-07-23

**Authors:** Zhe Wang, Zhiling Cheng, Yifan Zheng, Yang Liu, Zhihan Zhang, Yuan Gao, Congxin Li

**Affiliations:** 1 Department of Pharmacy, Hebei Medical University Third Hospital, Shijiazhuang, Hebei, China; 2 Hebei Medical University, Shijiazhuang, Hebei, China; 3 Department of Nutrition, The Fourth Hospital of Hebei Medical University, Shijiazhuang, China; 4 Hebei Provincial Key Laboratory of Precision Anesthesiology and Organ Protection, Shijiazhuang, China

**Keywords:** cardiovascular adverse events, major adverse cardiovascular events, meta-analysis, target, TKIs

## Abstract

**Objective:**

To assess the association between different tyrosine kinase inhibitors (TKIs) and the risk of major adverse cardiovascular events (MACE).

**Data sources:**

PubMed, the Cochrane Library, Embase, WanFang Data, and CNKI were searched for randomized controlled trials (RCTs) from inception to June 2026.

**Results:**

A total of 25 RCTs met the inclusion criteria, encompassing 9,068 patients. The risk of MACE was significantly higher in the TKI-treated group than in the control group (odds ratio [OR] = 2.13; 95% confidence interval [CI], 1.11–4.07; *P* = 0.02). The ORs for QT prolongation, hypertension, and arrhythmias were 6.05 (95% CI, 3.70–9.89; *P* < 0.00001), 4.18 (95% CI, 2.19–7.95; *P* < 0.0001), and 5.40 (95% CI, 3.43–8.50; *P* < 0.00001), respectively. Subgroup analysis indicated that epidermal growth factor receptor inhibitors (EGFR-TKIs) were associated with the highest risk of cardiovascular adverse events, followed by vascular endothelial growth factor receptor inhibitors (VEGFR-TKIs), platelet-derived growth factor receptor inhibitors (PDGFR-TKIs), and stem cell factor receptor inhibitors (c-Kit TKIs).

**Conclusion:**

TKI therapy significantly prolongs progression-free survival. However, the incidence of cardiovascular adverse events, including QT prolongation, hypertension, and arrhythmias, is notably higher with TKI use. EGFR-TKIs show the highest cardiovascular risk, followed by VEGFR-TKIs, PDGFR-TKIs, and c-Kit TKIs. Clinicians should be aware of these risks and ensure regular cardiovascular monitoring during treatment.

**Systematic Review Registration:**

https://www.crd.york.ac.uk/PROSPERO/view/CRD420251084805, identifier CRD420251084805.

## 1 Introduction

Tyrosine kinase inhibitors (TKIs) have substantially improved survival outcomes in patients with various malignancies by selectively targeting aberrant signaling pathways that are critical for tumor cell proliferation, differentiation, and apoptosis (Whittaker et al., 2010; Paul and Mukhopadhyay, 2004). However, the cardiovascular toxicity associated with TKI therapy has raised increasing concern (Van Leeuwen et al., 2020; Lenihan and Kowey, 2013; Chang et al., 2023; Chaar et al., 2018). Beyond their primary targets, many TKIs inhibit multiple kinases to enhance therapeutic efficacy, which may inadvertently increase the risk of major adverse cardiovascular events (MACEs). Existing studies have demonstrated substantial variation in the cardiotoxicity profiles of different TKIs, with distinct patterns of MACEs depending on the specific agent used (Shi et al., 2025).

Beyond the field of oncology, the therapeutic potential of TKIs is being increasingly investigated across a wide range of human diseases. In the context of neurodegenerative disorders, strategies involving multitarget kinase inhibition have been explored in Alzheimer’s disease (AD), where kinase inhibitors that target the processing of amyloid precursor protein and the phosphorylation pathways of tau proteins have shown promise (Tripathi et al., 2025). In the realm of inflammatory and autoimmune diseases, the selective inhibition of tyrosine kinase 2 (TYK2) has emerged as an innovative therapeutic strategy, providing targeted modulation of immune responses (Rzeczycki et al., 2025). In the treatment of non-small cell lung cancer (NSCLC), TKIs that target mutations in the EGFR, rearrangements in anaplastic lymphoma kinase (ALK)/ROS proto-oncogene 1 (ROS1)/rearranged during transfection (RET), and amplification of the mesenchymal-epithelial transition factor (MET) have significantly improved clinical outcomes. However, the development of acquired resistance mechanisms, such as histologic transformation to sarcomatoid carcinoma, continues to pose a significant challenge (Pang et al., 2024). In the context of advanced clear cell renal cell carcinoma, the integration of VEGFR-TKIs with immune checkpoint inhibitors (ICIs) has fundamentally transformed first-line treatment strategies, demonstrating substantial therapeutic efficacy (Xu et al., 2026). Furthermore, computational analyses have proposed that specific EGFR-targeting scaffolds may possess inhibitory activity against the human ether-à-go-go-related gene II (hERG II), suggesting a potential electrophysiological mechanism underlying the arrhythmogenic risk associated with TKIs, which merits further exploration (Rajaraman et al., 2025). Despite these therapeutic advancements, the increasing application of TKIs across various disease contexts has uncovered a range of cardiovascular toxicities, with variability in frequency and severity observed across different drug classes and individual agents (Zheng, 2025).

This study focused on 10 TKIs, namely, gefitinib, erlotinib, lenvatinib, vandetanib, cabozantinib, axitinib, pazopanib, sunitinib, sorafenib, and osimertinib, to evaluate differences in cardiovascular toxicity among these agents and to explore potential associations between their molecular targets and cardiovascular risk (Chang et al., 2023). We systematically screened RCTs assessing the safety and efficacy of these TKIs and performed a meta-analysis to compare their safety profiles. Particular attention was given to MACEs associated with each TKI, with the goal of providing evidence-based guidance for clinical decision-making.

## 2 Materials and methods

### 2.1 Study protocol and registration

This study followed a prespecified protocol and was registered in the International Prospective Register of Systematic Reviews (PROSPERO; registration number CRD420251084805). The Preferred Reporting Items for Systematic Reviews and Meta-Analyses (PRISMA) guidelines were followed throughout the study.

### 2.2 Search strategy

We searched PubMed, the Cochrane Library, Embase, WanFang Data, and CNKI for studies evaluating the cardiovascular toxicity of TKIs. The search terms included “gefitinib,” “erlotinib,” “lenvatinib,” “vandetanib,” “cabozantinib,” “axitinib,” “pazopanib,” “sunitinib,” “sorafenib,” “osimertinib,” “placebo,” “cardiac,” “CVAE,” “VEGFR-TKI,” “VEGFR,” “EGFR-TKI,” “EGFR,” “cardiotoxicity,” “cardiac arrest,” “heart failure,” “QT prolongation,” “myocardial ischemia,” “hypertension,” “arrhythmia,” “MACE,” and “cardiovascular events.”

### 2.3 Inclusion and exclusion criteria

#### 2.3.1 Inclusion criteria

(1) RCTs published from database inception through June 2026; (2) trials comparing one of the selected TKIs (experimental group) with placebo (control group) for the treatment of any cancer type. The use of other standard treatments in combination with TKIs or placebo was permitted, and drug dosages in both groups were not restricted. For regimens consisting of a TKI plus another active agent, the control arm was required to receive identical background therapy plus placebo or no TKI; (3) trials reporting sufficient information on primary and secondary outcomes, as well as baseline characteristics; and (4) studies involving adults aged 18 years or older, with no restrictions on sex, race, or nationality.

#### 2.3.2 Outcome measures

The primary outcome was MACE, defined in accordance with the standard three-point criteria jointly recognized by the United States Food and Drug Administration (FDA) and the European Medicines Agency (EMA), which includes non-fatal myocardial infarction, non-fatal stroke, and cardiovascular mortality. Secondary outcomes included other individual cardiovascular adverse events specifically QT interval prolongation, hypertension, arrhythmia, and heart failure which were analyzed separately to characterize the broader cardiovascular safety profile of each TKI class. In addition, efficacy outcomes, namely, overall survival (OS) and progression-free survival (PFS), were also assessed as secondary endpoints to provide a comprehensive benefit-risk evaluation.

#### 2.3.3 Exclusion criteria

(1) Duplicate publications; (2) publication types such as reviews, letters, comments, editorials, and case reports; (3) studies for which the full text was unavailable; (4) non-randomized studies, including retrospective studies, case–control studies, and conference abstracts; (5) studies with no extractable data, such as those reporting only graphical results without numerical data and for which no response was obtained from the corresponding authors; and (6) studies with unclear intervention comparability. For regimens consisting of a TKI plus an active agent, the control arm was required to receive identical background therapy plus placebo or no TKI. Studies with any differences in background therapy were excluded.

### 2.4 Literature screening and data extraction

Two researchers (Zhe Wang and Zhiling Cheng) independently screened the literature according to the inclusion and exclusion criteria. Any disagreements were resolved through discussion or, when necessary, by consultation with a third reviewer (Congxin Li). Extracted data included the first author, year of publication, sample size, study region, patient race, general patient characteristics, baseline characteristics, trial phase, follow-up duration, and outcome measures.

### 2.5 Quality assessment of included studies

The quality of the included studies was assessed using the Cochrane Risk of Bias Tool (Version 1). Two researchers independently evaluated the methodological quality of each study, and any disagreements were resolved through discussion or consultation with a third reviewer. The tool assesses five domains: the randomization process, deviations from intended interventions, missing outcome data, outcome measurement, and selective reporting. The risk of bias in each domain was classified as low, unclear, or high.

### 2.6 Statistical methods

This meta-analysis was performed using Review Manager 5.4 and Stata 18.0. For dichotomous outcomes, odds ratios (ORs) with 95% confidence intervals (CIs) were calculated. For time-to-event outcomes, hazard ratios (HRs) with 95% CIs were used. Because ORs were used as safety indicators and HRs as efficacy indicators, values of OR < 1 and HR < 1 were considered favorable for the intervention. Statistical heterogeneity among studies was assessed using the χ^2^ test, with *P* < 0.10 indicating significant heterogeneity. A random-effects model was used for the overall analysis, whereas fixed-effects models were applied in subgroup analyses.

Sensitivity analyses were conducted by sequentially excluding individual studies to assess their influence on the overall results. Funnel plots were used as the primary visual tool for evaluating publication bias and asymmetry, and Egger’s test was employed as the primary quantitative method. The results of Begg’s test are also reported as Supplementary Material; however, the principal conclusions regarding publication bias were based on Egger’s test.

## 3 Results

### 3.1 Literature search results

**FIGURE 1:**
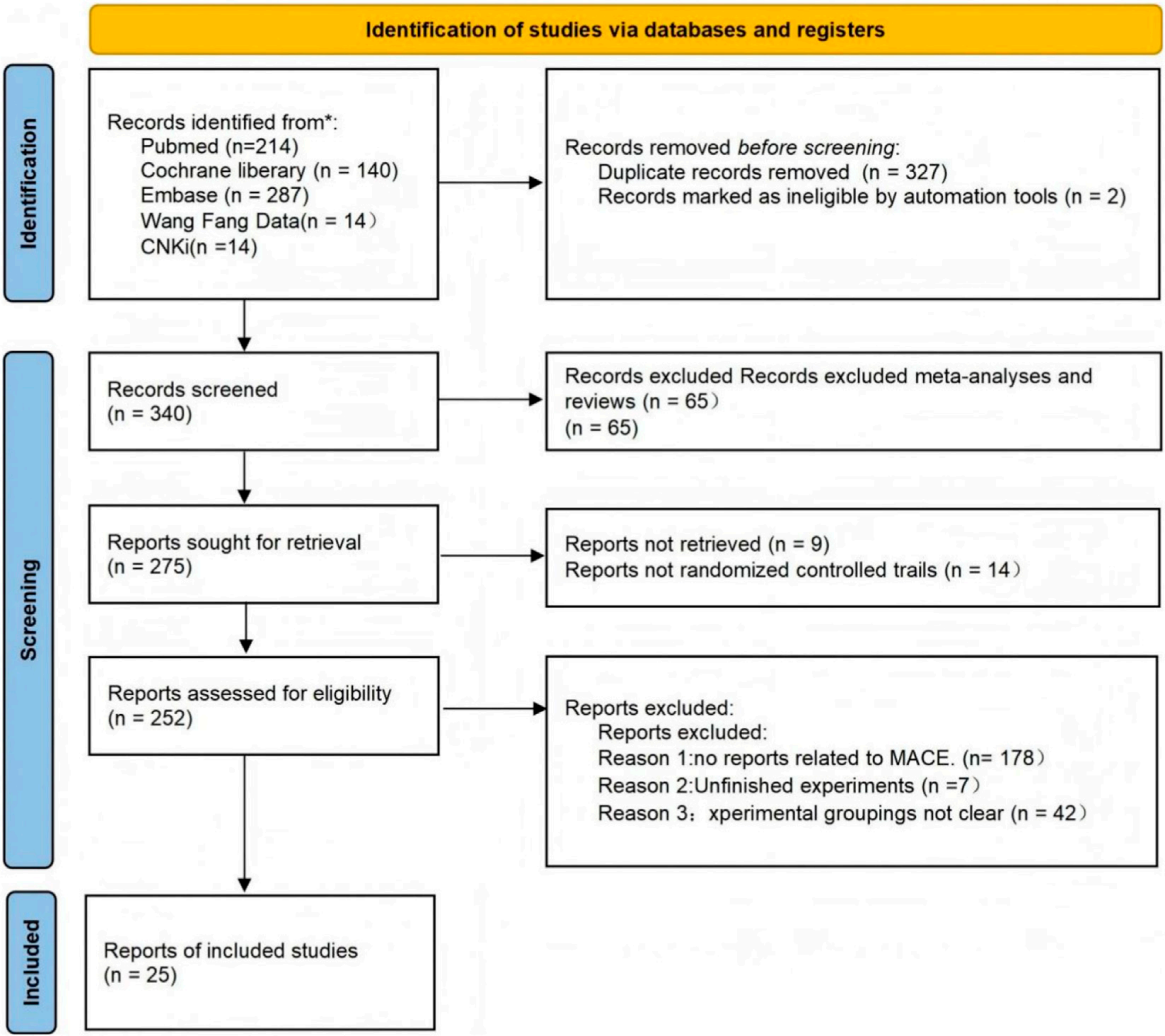
PRISMA 2020 flow diagram of the study selection process. The flow diagram summarizes the study selection process according to the PRISMA 2020 guidelines. The numbers of records identified through database searching, duplicates removed, records screened, full-text articles assessed for eligibility, and studies ultimately included are shown. Reasons for exclusion at the full-text stage are provided.

The search of studies published through June 2026 initially identified 667 relevant articles. After screening titles, abstracts, and full texts, 25 studies were included in the final analysis (Gross-Goupil et al., 2018; Kelly et al., 2015; Johnson et al., 2013; Cicènas et al., 2016; Brose et al., 2024; Middleton et al., 2017; Gupta et al., 2016; Sanborn et al., 2017; Argiris et al., 2013; Soria et al., 2015; Dutton et al., 2014; Cristofanilli et al., 2010; Brose et al., 2021; Brose et al., 2022; Matsubara et al., 2024; Yang et al., 2024; Zheng et al., 2021; Sherman et al., 2023; Gonçalves et al., 2012; Schwartzberg et al., 2013; Haas et al., 2015; Kang and Hu, 2016; Herbst et al., 2023; Lu et al., 2024; Chan et al., 2025). A total of 9,361 patients were enrolled, including 5,233 in the experimental group and 4,128 in the control group. Twenty-four studies were published in English, and one was published in Chinese. The treatment regimens varied across studies: 1 evaluated axitinib, 3 erlotinib, 5 vandetanib, 4 gefitinib, 3 cabozantinib, 3 lenvatinib, 1 pazopanib, 3 sunitinib, 5 sorafenib, and 2 osimertinib. In all studies, the control group received placebo. The literature screening process is presented in Figure 1. The search strategies are summarized in Supplementary Table S1, and the baseline characteristics of the included studies are summarized in Table 1.

**TABLE 1:** Basic characteristics of the included studies.

Study	Group	Treatment (mg/day)	Patients	Race	Age (years)	MACE report	mPFS (months)	mOS (months)	Phase	Disease
Gross-Goupil 2018 (Gross-Goupil et al., 2018)	Axitinib	10	280	White/Black/Asian/Other	58.0 ± 7.0	1	NR	NR	III	RCC
	Placebo	-	250		58.0 ± 7.0	1	NR	NR	​	​
Kelly 2015 (Kelly et al., 2015)	Erlotinib	150	611	White/Black/Asian/Other	62.0 ± 9.28	2	NR	NR	III	NSCLC
	Placebo	-	343		61.8 ± 9.34	0	NR	NR	​	​
Johnson 2013 (Johnson et al., 2013)	Erlotinib	150	370	White/Black/Asian/Other	64	31	4.8	14.4	III	NSCLC
	Placebo	-	373		64	29	3.7	13.3	​	​
Cicènas 2016 (Cicènas et al., 2016)	Erlotinib	150	322	White/Asian/Other	61 (30–86)	2	3.0	9.7	III	NSCLC
	Placebo	-	321		61 (26–81)	0	2.8	9.5	​	​
Brose 2024 (Brose et al., 2024)	Vandetanib	300	117	White/Black/Asian/Other	64.2 ± 9.69	36	10.0	NR	III	Thyroid cancer
	Placebo	-	118		63.2 ± 11.0	4	5.7	NR	​	​
Gary 2017 (Middleton et al., 2017)	Vandetanib	300	72	White/Black/Asian/Other	66.5 (61–73)	23	NR	8.83	II	ViP
	Placebo	-	70		67.5 (61–73)	2	NR	8.95	​	​
Avinash 2016 (Gupta et al., 2016)	Vandetanib	300	10	White/Black/Asian/Other	57 (42–77)	4	3.3	4.6	II	Melanoma
	Placebo	-	8		64 (44–83)	0	2.5	2.5	​	​
Sanborn 2017 (Sanborn et al., 2017)	Vandetanib	100	40	White/Black/Asian	64 (47–74)	5	NR	13.24	II	SCLC
	Placebo	-	33		63 (35–90)	1	NR	9.23	​	​
Argiris 2013 (Argiris et al., 2013)	Gefetinib	250	124	White/Other	61.4 (28.0–86.5)	1	NR	7.3	III	R/M SCCHN
	Placebo	-	129		60.8 (41.6–84.4)	2	NR	6.0	​	​
Soria 2015 (Soria et al., 2015)	Gefetinib	250	133	White/Black/Asian/Other	60 (33–79)	0	NR	NR	III	NSCLC
	Placebo	-	132		58 (35–79)	1	NR	NR	​	​
Dutton 2014 (Dutton et al., 2014)	Gefetinib	500	224	White/Black/Asian/Other	67.7 (58.0–70.1)	6	1.57	3.73	III	COG
	Placebo	-	225		64.9 (58.2–70.7)	4	1.17	3.63	​	​
Cristofanilli 2010 (Cristofanilli et al., 2010)	Gefetinib	250	43	Caucasian/Black/Other	61 (41–82)	1	14.7	NR	II	MBC
	Placebo	-	50		58 (37–84)	0	8.4	NR	​	​
Marcia 2021 (Brose et al., 2021)	Cabozantinib	60	125	White/Black/Asian/Other	65 (56–72)	3	NR	NR	III	DTC
	Placebo	-	62		66 (56–72)	1	1.9	NR	​	​
Brose 2022 (Brose et al., 2022)	Cabozantinib	60	178	White/Black/Asian/Other	65 (31–85)	2	11.0	NR	III	RAIR-DTC
	Placebo	-	88		66 (37–87)	0	1.9	NR	​	​
Nobuaki 2023 (Matsubara et al., 2024)	Lenvatinib	20	245	White/Black/Asian/Other	74 (66–79)	1	4.5	11.8	III	UC
	Placebo	-	242		73 (67–78)	0	4.0	12.9	​	​
James 2023 (Yang et al., 2024)	Lenvatinib	20	309	Asian/Other	66 (34–85)	20	6.6	14.1	III	NSCLC
	Placebo	-	314		66 (37–87)	8	4.2	16.4	​	​
Zheng 2023 (Zheng et al., 2021)	Lenvatinib	24	103	Asian	61.0 (28–80)	1	23.9	NR	III	NSCLC
	Placebo	-	48		60.0 (22–80)	0	3.7	NR	​	​
Eric 2023 (Sherman et al., 2023)	Pazopanib	400	36	White/Black/Asian/Other	62.4	1	NR	5.7	II	ATC
	Placebo	-	35		62.6	2	NR	7.3	​	​
Gongalves 2012 (Gonçalves et al., 2012)	Sorafenib	800	50	White/Black/Asian/Other	64 (40–82)	4	3.8	8	III	Pancreatic cancer
	Placebo	-	52		61 (42–85)	6	5.7	9.2	​	​
Schwartzberg 2013 (Schwartzberg et al., 2013)	Sorafenib	800	81	White/Black/Asian/Other	53.5 ± 10.6	1	3.4	13.4	II	Breast Cancer
	Placebo	-	79		54.2 ± 11.0	0	2.7	11.4	​	​
Haas 2015 (Haas et al., 2015)	Sorafenib	800	510	White/Black/Asian/Other	55 (48–63)	56	NR	NR	III	RCC
	Sunitinib	50	513		56 (49–64)	59	NR	NR	​	​
	Placebo	-	580		57 (49–64)	48	NR	NR	​	​
Kang 2015 (Kang and Hu, 2016)	Vandetanib	100	62	Asian	75.01 ± 2.9	5	6.1	8.7	II	NSCLC
	Placebo	-	62		75.58 ± 3.1	2	5.6	10.1	​	​
Herbst 2023 (Herbst et al., 2023)	Osimertinib	80	337	White/Asian/Other	62.5 ± 10.3	30	NR	NR	III	NSCLC
	Placebo	-	343		61.6 ± 10.5	8	NR	NR	​	​
Lu 2024 (Lu et al., 2024)	Osimertinib	80	143	Asian/Other	62 (36–84)	8	39.1	NR	III	NSCLC
	Placebo	-	73		64 (37–83)	1	5.6	NR	​	​
Chan 2025 (Chan et al., 2025)	Cabozantinib	60	195	White/Black/Asian/Other	63 (28–86)	42	8.4	NR	III	NET
	Placebo	-	98		65 (30–82)	5	3.9	NR		

NR, not reported; mPFS, median progression-free survival; mOS, median overall survival; RCC, renal cell carcinoma; NSCLC, non-small cell lung cancer; VIP, vasoactive intestinal peptide-secreting tumor; SCLC, small cell lung cancer; R/M SCCHN, recurrent/metastatic squamous cell carcinoma of the head and neck; COG, cholangiocarcinoma; MBC, metastatic breast cancer; DTC, differentiated thyroid carcinoma; RAIR-DTC, radioiodine-refractory differentiated thyroid carcinoma; UC, urothelial carcinoma; ATC, anaplastic thyroid carcinoma; NET, neuroendocrine tumors. Data for age are presented as either mean ± standard deviation (SD) or median (range), as reported in the original studies.

### 3.2 Assessment of study quality

All 25 studies were randomized controlled trials and used double-blind randomization methods. However, the presence of other potential sources of bias remained unclear. The quality assessment results are presented in Supplementary Figures S1, S2 in the Supplement.

### 3.3 Meta-analysis results

#### 3.3.1 Cardiovascular outcomes

##### 3.3.1.1 MACEs

**FIGURE 2:**
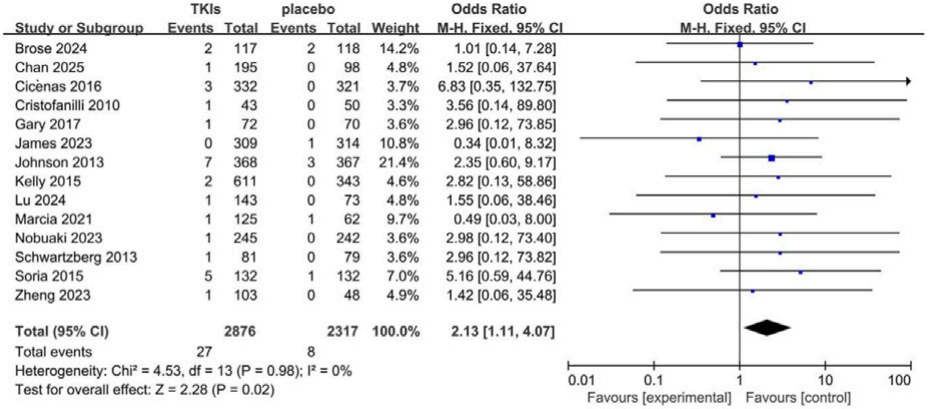
Forest plot of MACEs comparing tyrosine kinase inhibitors (TKIs) with placebo in patients with advanced malignancies. For MACEs, a fixed-effects model was used (I^2^ = 0%, *P* = 0.98). Squares represent the effect estimate (odds ratio [OR]) for each individual study; the size of each square is proportional to the weight assigned to that study in the meta-analysis. Horizontal lines indicate the 95% confidence interval (CI) for each study. The diamond at the bottom of the forest plot represents the pooled effect estimate; the width of the diamond corresponds to the 95% CI of the pooled estimate. Abbreviations: TKI, tyrosine kinase inhibitor; CI, confidence interval; OR, odds ratio; MACEs, major adverse cardiovascular events.

Fourteen studies reported MACEs. Significant heterogeneity was observed among the studies (*P* = 0.98, I^2^ = 0%); therefore, a fixed-effects model was applied. The pooled analysis showed that the risk of MACEs was significantly higher in the experimental group than in the control group (OR = 2.13; 95% CI, 1.11–4.07; *P* = 0.02). The results are presented in Figure 2.

##### 3.3.1.2 Subgroup analysis by drug target

We screened the therapeutic targets of the 10 selected TKIs (Supplementary Table S2) and summarized them using a Venn diagram (Supplementary Figure S3). Four common targets were identified across the 10 agents: EGFR, VEGFR, PDGFR, and c-Kit.

**FIGURE 3:**
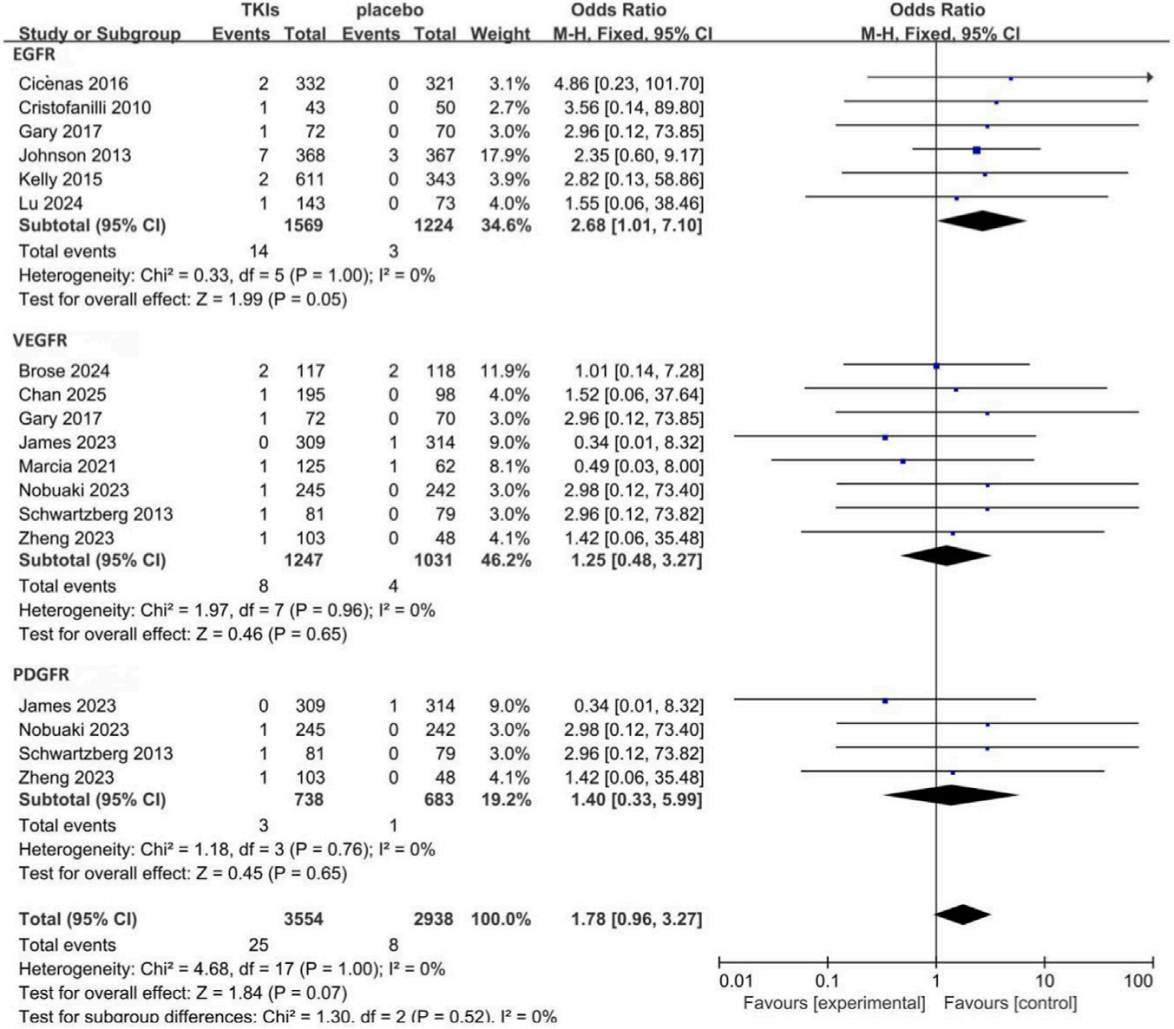
Subgroup analysis of MACE risk by TKI target class (EGFR-TKIs, VEGFR-TKIs, PDGFR-TKIs) compared with placebo. For subgroup analysis, a fixed-effects model was used (EGFR: I^2^ = 0%, *P* = 0.05; VEGFR: I^2^ = 0%, *P* = 0.65; PDGFR: I^2^ = 0%, *P* = 0.65). Squares represent the effect estimate (OR) for each individual study; the size of each square is proportional to the weight assigned to that study in the meta-analysis. Horizontal lines indicate the 95% CI for each study. The diamond at the bottom of each forest plot represents the pooled effect estimate; the width of the diamond corresponds to the 95% CI of the pooled estimate. Abbreviations: TKI, tyrosine kinase inhibitor; EGFR, epidermal growth factor receptor; VEGFR, vascular endothelial growth factor receptor; PDGFR, platelet-derived growth factor receptor; CI, confidence interval; OR, odds ratio; MACEs, major adverse cardiovascular events.

Subgroup analysis based on drug targets indicated that EGFR-TKIs (gefitinib, erlotinib, vandetanib, and osimertinib) were associated with a significantly higher risk of MACEs (OR = 2.68; 95% CI, 1.01–7.10; *P* = 0.05). VEGFR-TKIs (lenvatinib, vandetanib, cabozantinib, axitinib, pazopanib, sunitinib, and sorafenib), PDGFR-TKIs (lenvatinib, axitinib, pazopanib, sunitinib, and sorafenib) showed no significant differences (P = 0.65). These findings are shown in Figure 3.

Comparison of ORs across the four target categories revealed that EGFR-TKIs carried the highest risk of MACEs.

##### 3.3.1.3 Subgroup analysis by single-target vs. multi-target TKIs

**FIGURE 4:**
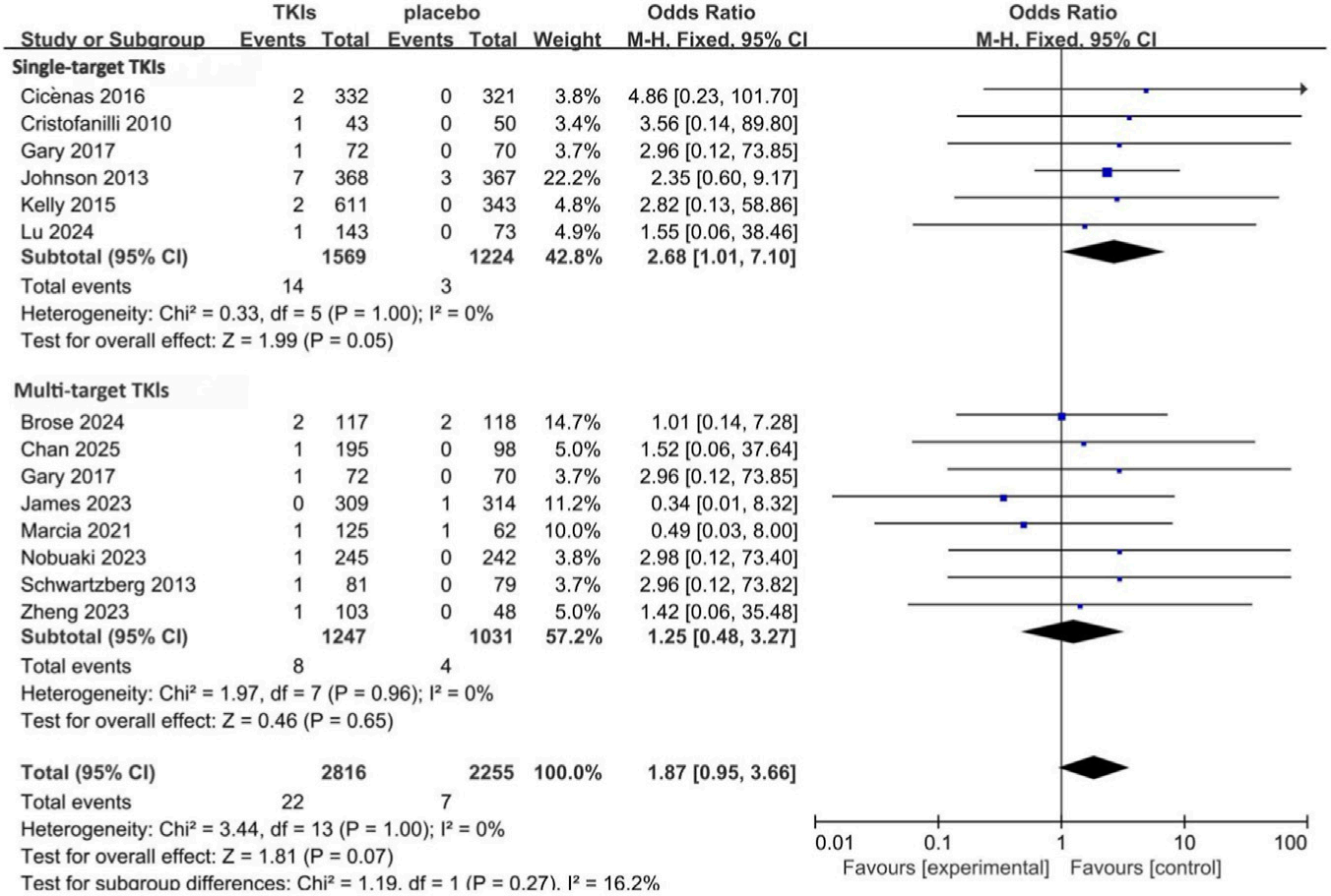
Subgroup analysis of MACE risk comparing single-target TKIs and multi-target TKIs, with placebo as the common reference. For subgroup analysis, a fixed-effects model was used (single-target TKIs: I^2^ = 0%, *P* = 0.05; multi-target TKIs: I^2^ = 0%, *P* = 0.65). Squares represent the effect estimate (OR) for each individual study; the size of each square is proportional to the weight assigned to that study in the meta-analysis. Horizontal lines indicate the 95% CI for each study. The diamond at the bottom of each forest plot represents the pooled effect estimate; the width of the diamond corresponds to the 95% CI of the pooled estimate. Abbreviations: TKI, tyrosine kinase inhibitor; CI, confidence interval; OR, odds ratio; MACEs, major adverse cardiovascular events.

A subgroup analysis comparing single-target TKIs with multi-target TKIs was performed. Single-target TKIs included gefitinib, erlotinib, and osimertinib, whereas multi-target TKIs included lenvatinib, vandetanib, cabozantinib, axitinib, pazopanib, sunitinib, and sorafenib. The meta-analysis demonstrated that single-target TKIs were associated with a significantly higher risk of MACEs than the control group (OR = 2.68; 95% CI, 1.01–7.10; *P* = 0.05). Multi-target TKIs also showed no significant differencres (P = 0.65). When comparing ORs between the two categories, the risk of MACEs was higher with single-target TKIs than with multi-target TKIs. The results are presented in Figure 4.

#### 3.3.2 Analysis of different types of cardiovascular adverse events (CVAEs)

In addition to the components included in the MACE definition, we also selected other CVAEs to enable a broader and more comprehensive assessment of the cardiovascular toxicity profile of TKIs. We reviewed the product labels of the selected TKIs and summarized the types of CVAEs associated with these agents. The CVAEs reported in the prescribing information are summarized in Supplementary Table S3.

##### 3.3.2.1 CVAEs

Nineteen studies reported CVAEs. Significant heterogeneity was observed among the studies (*P* = 0.003, I^2^ = 52%); therefore, a random-effects model was applied. The pooled analysis showed that the risk of CVAEs was significantly higher in the experimental group than in the control group (OR = 3.11; 95% CI, 2.05–4.71; *P* < 0.00001). The results are presented in Supplementary Figure S4.

Subgroup analysis based on drug targets indicated that EGFR-TKIs (gefitinib, erlotinib, vandetanib, and osimertinib) were associated with a significantly higher risk of CVAEs (OR = 5.87; 95% CI, 3.73–9.23; *P* < 0.00001). VEGFR-TKIs (lenvatinib, vandetanib, cabozantinib, axitinib, pazopanib, sunitinib, and sorafenib) also demonstrated a significantly increased risk (OR = 2.35; 95% CI, 1.88–2.93; *P* < 0.0001). For PDGFR-TKIs (lenvatinib, axitinib, pazopanib, sunitinib, and sorafenib), the risk of CVAEs remained significantly higher than that in the control group (OR = 1.62; 95% CI, 1.26–2.09; *P* = 0.001). Similarly, c-Kit TKIs (pazopanib and sorafenib) were associated with a significantly increased risk of CVAEs compared with the control group (OR = 1.56; 95% CI, 1.07–2.26; *P* = 0.02). These findings are shown in Supplementary Figure S5. Comparison of ORs across the four target categories revealed that EGFR-TKIs carried the highest risk of CVAEs, followed by VEGFR-TKIs, PDGFR-TKIs, and c-Kit TKIs.

##### 3.3.2.2 Individual adverse events

QT prolongation was the most commonly reported CVAE, with seven drugs associated with QT prolongation risk: gefitinib, lenvatinib, vandetanib, pazopanib, sunitinib, sorafenib, and osimertinib. We also summarized the CVAEs reported in the included RCTs. QT prolongation and heart failure were the most frequently documented events, whereas other CVAEs (including cardiac arrest and myocardial ischemia) were reported less often. Accordingly, we focused our analysis on QT prolongation, heart failure, hypertension, and arrhythmias.

**FIGURE 5:**
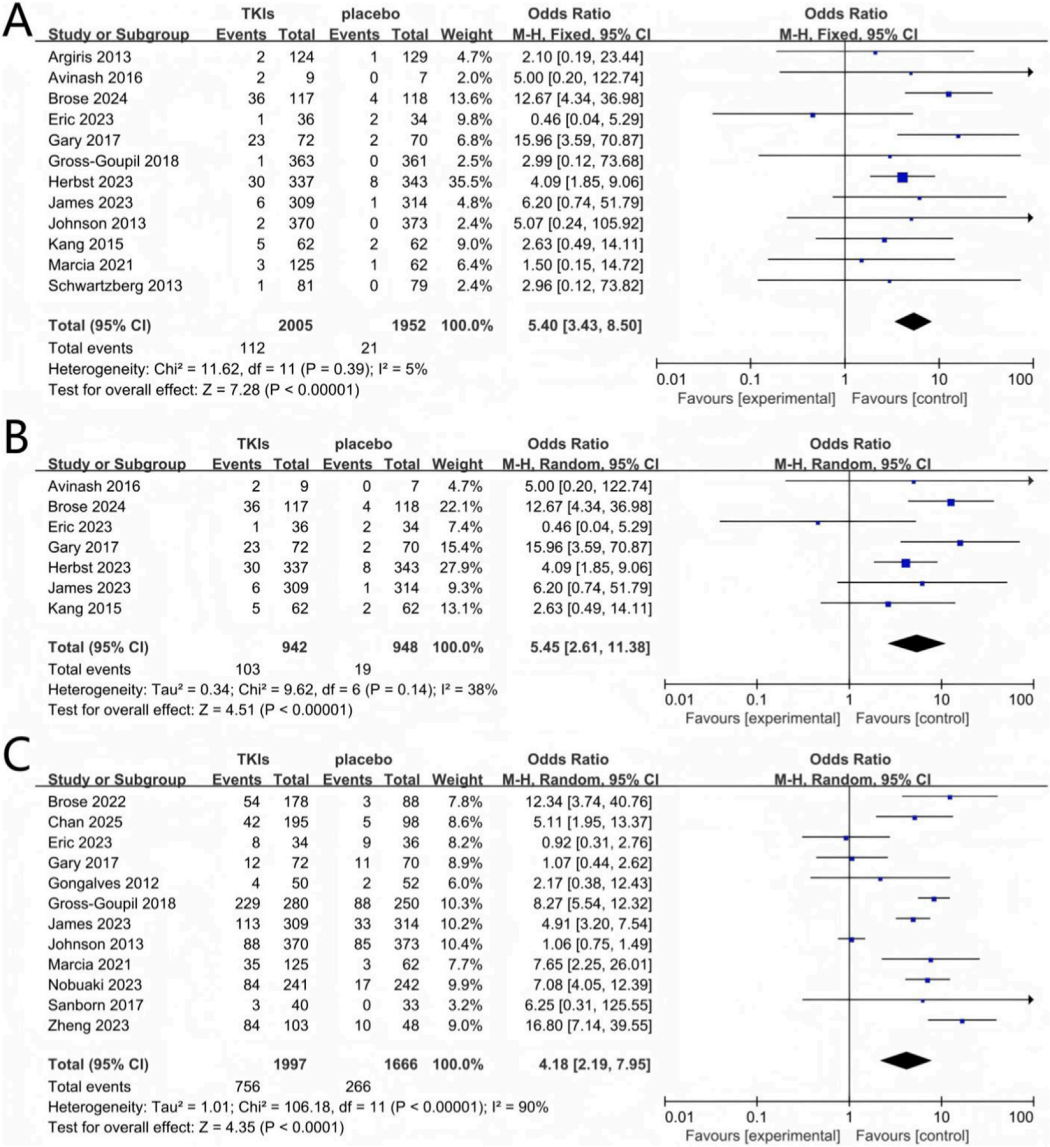
Forest plots of CVAEs associated with TKIs compared with placebo: **(A)** arrhythmias, **(B)** QT interval prolongation, and **(C)** hypertension. For arrhythmias, a fixed-effects model was used (I^2^ = 5%, *P* < 0.00001). For QT interval prolongation, a random-effects model was used (I^2^ = 38%, *P* < 0.00001). For hypertension, a random-effects model was used (I^2^ = 90%, *P* < 0.0001). Squares represent the effect estimate (OR) for each individual study; the size of each square is proportional to the weight assigned to that study in the meta-analysis. Horizontal lines indicate the 95% CI for each study. The diamond at the bottom of each forest plot represents the pooled effect estimate; the width of the diamond corresponds to the 95% CI of the pooled estimate. Abbreviations: TKI, tyrosine kinase inhibitor; CI, confidence interval; OR, odds ratio; CVAEs, cardiovascular adverse events.

Twelve studies reported arrhythmias. The pooled analysis indicated that the risk of arrhythmias was significantly higher in the experimental group than in the control group (OR = 5.40; 95% CI, 3.43–8.50; *P* < 0.00001). The results are shown in Figure 5A. Subgroup analysis by drug target demonstrated that EGFR-TKIs were associated with a significantly higher risk of arrhythmias than the control group (OR = 8.71; 95% CI, 4.43–17.13; *P* < 0.00001). VEGFR-TKIs also showed an elevated risk (OR = 6.49; 95% CI, 3.62–11.65; *P* < 0.00001). For PDGFR-TKIs and c-Kit TKIs, no significant differences were observed (*P* = 0.12 and *P* = 0.96, respectively). The results are presented in Supplementary Figure S1.

Seven studies reported QT prolongation. No significant heterogeneity was observed (*P* = 0.14, I^2^ = 38%); therefore, a random-effects model was used. The experimental group had a significantly higher risk of QT prolongation than the control group (OR = 5.45; 95% CI, 2.61–11.38; *P* < 0.00001). The results are shown in Figure 5B. Subgroup analyses were conducted according to drug targets. QT prolongation–related RCTs involved EGFR-TKIs and VEGFR/PDGFR-TKIs. EGFR-TKIs showed a significantly increased risk (OR = 6.64; 95% CI, 3.96–11.14; *P* < 0.00001), whereas VEGFR/PDGFR-TKIs showed no significant difference compared with the control group (*P* = 0.22). The results are shown in Supplementary Figure S7.

Twelve studies reported hypertension. Significant heterogeneity was detected (*P* < 0.0001, I^2^ = 90%), leading to the use of a random-effects model. The pooled analysis showed a higher risk of hypertension in the experimental group than in the control group (OR = 4.18; 95% CI, 2.19–7.95; *P* < 0.00001). The results are presented in Figure 5C. Subgroup analysis showed no significant difference in hypertension risk for EGFR-TKIs (*P* = 0.57). In contrast, VEGFR-TKIs showed a significantly increased risk (OR = 5.83; 95% CI, 4.71–7.21; *P* < 0.00001), as did PDGFR-TKIs (OR = 6.23; 95% CI, 4.92–7.90; *P* < 0.00001). No significant difference was found for c-Kit TKIs (*P* = 0.71). The results are shown in Supplementary Figure S8

Six studies reported heart failure. No significant difference was observed between groups, and subgroup analyses based on therapeutic targets also showed no significant findings. The results are presented in Supplementary Figure S9.

##### 3.3.2.3 Cardiogenic death

Ten studies reported cardiac-related death, and no significant difference was observed between the experimental and control groups (*P* = 0.22). Subgroup analyses according to therapeutic target also showed no significant differences. The results are presented in Supplementary Figures S10, S11.

#### 3.3.3 Analysis of CVAEs in different tumor types

For the subgroup analysis by tumor type, trials were categorized into two groups: NSCLC and other solid tumors, which included all remaining studies. The “other solid tumors” category aggregated studies with limited sample sizes.

Eight studies reported CVAEs in NSCLC. The pooled results indicated that the risk of CVAEs was significantly higher in the experimental group than in the control group (OR = 2.11; 95% CI, 1.22–3.67; *P* = 0.008). The results are shown in Supplementary Figure S12. Subgroup analysis by drug target demonstrated that EGFR-TKIs were significantly associated with a higher risk of CVAEs compared with the control group (OR = 3.41; 95% CI, 1.79–6.49; *P* = 0.0002). VEGFR-TKIs also showed an elevated risk (OR = 2.60; 95% CI, 1.27–5.33; *P* = 0.009). When comparing ORs between the two categories, the risk of MACEs was higher with EGFR-TKIs than with VEGFR-TKIs. The results are presented in Supplementary Figure S13.

Fourteen studies reported CVAEs in other solid tumors. The pooled results indicated that the risk of CVAEs was significantly higher in the experimental group than in the control group (OR = 3.36; 95% CI, 1.94–5.84; *P* < 0.0001). The results are shown in Supplementary Figure S14. Subgroup analysis based on drug targets indicated that EGFR-TKIs were significantly associated with a higher risk of CVAEs (OR = 10.36; 95% CI, 5.20–20.66; *P* < 0.00001). VEGFR-TKIs also demonstrated a significantly increased risk (OR = 2.32; 95% CI, 1.84–2.93; *P* < 0.0001). For PDGFR-TKIs, the risk of CVAEs remained higher in the experimental group than in the control group (OR = 1.53; 95% CI, 1.17–2.00; *P* = 0.002). For c-Kit TKIs, the risk of CVAEs also remained higher in the experimental group than in the control group (OR = 1.56; 95% CI, 1.07–2.26; *P* = 0.02). Comparison of ORs across the four target categories revealed that EGFR-TKIs carried the highest risk of CVAEs in other solid tumors, followed by VEGFR-TKIs, C-kit-TKIs, and PDGFR-TKIs. The results are presented in Supplementary Figure S15.

#### 3.3.4 Efficacy outcomes

##### 3.3.4.1 OS

Thirteen studies reported OS outcomes. There was no significant statistical heterogeneity among the studies (*P* = 0.28, I^2^ = 16%); therefore, a fixed-effects model was applied. The pooled analysis showed no significant difference in OS between the experimental and control groups (HR = 1.00; 95% CI, 0.92–1.08; *P* = 0.93). The results are presented in Supplementary Figure S16.

##### 3.3.4.2 PFS

Fourteen studies reported PFS. Significant statistical heterogeneity was observed among the studies (*P* < 0.00001, I^2^ = 90%); therefore, a random-effects model was applied. The pooled results showed that patients in the experimental group had significantly longer PFS than those in the control group (HR = 0.59; 95% CI, 0.45–0.75; *P* < 0.0001). The results are presented in Supplementary Figure S17.

##### 3.3.4.3 Subgroup analysis of PFS by drug target

Subgroup analysis of PFS based on drug targets showed that EGFR-TKIs resulted in significantly longer PFS in the experimental group than in the control group (HR = 0.87; 95% CI, 0.79–0.96). For VEGFR-TKIs, the experimental group also demonstrated significantly longer PFS (HR = 0.64; 95% CI, 0.58–0.71). For PDGFR-TKIs, the experimental group likewise demonstrated significantly longer PFS (HR = 0.68; 95% CI, 0.59–0.79). However, for c-Kit TKIs, no significant difference in PFS was observed between the experimental and control groups. These results are shown in Supplementary Figure S18.

To compare the magnitude of PFS improvement among the four targets, HRs were analyzed; a smaller HR indicates greater PFS prolongation. Among the four target classes, VEGFR-TKIs demonstrated the greatest improvement in PFS.

#### 3.3.5 Sensitivity analysis and publication bias

Sensitivity analysis was performed using MACEs as the outcome measure. The results indicated no significant change in the overall effect size after the sequential exclusion of individual studies (*P* > 0.05). However, two studies exhibited a notable influence on the effect estimates (Supplementary Figure S19). Funnel plot analysis showed no apparent asymmetry (Supplementary Figure S20). Egger’s test (*P* = 0.095) indicated a low likelihood of publication bias, which was supported by Begg’s test (*P* = 0.965) as a supplementary analysis.

## 4 Discussion

With the advancement of molecular-targeted therapies, TKIs have become increasingly important in cancer treatment (Yang et al., 2022). In 2001, the U.S. Food and Drug Administration approved imatinib as the first TKI, marking the beginning of the TKI era. Over the past two decades, numerous additional TKIs have been developed and widely applied in clinical practice (Jabbour and Kantarjian, 2024). However, these agents are frequently associated with substantial cardiovascular toxicity, particularly multi-target TKIs (Rathmell et al., 2022; Meillan et al., 2025; Zhang et al., 2025). In clinical settings, determining the most appropriate TKI for patients, especially those with pre-existing cardiovascular disease, remains a major challenge and an important area of ongoing research (Nogueira-Ferreira et al., 2022; Yang and Bu, 2016; Li et al., 2021). To address this need, the present study systematically evaluated the cardiovascular safety of 10 TKIs with known cardiotoxic potential and secondarily assessed their efficacy by analyzing data from RCTs (Gross-Goupil et al., 2018; Kelly et al., 2015; Johnson et al., 2013; Cicènas et al., 2016; Brose et al., 2024; Middleton et al., 2017; Gupta et al., 2016; Sanborn et al., 2017; Argiris et al., 2013; Soria et al., 2015; Dutton et al., 2014; Cristofanilli et al., 2010; Brose et al., 2021; Brose et al., 2022; Matsubara et al., 2024; Yang et al., 2024; Zheng et al., 2021; Sherman et al., 2023; Gonçalves et al., 2012; Schwartzberg et al., 2013; Haas et al., 2015; Kang and Hu, 2016; Herbst et al., 2023; Lu et al., 2024; Chan et al., 2025).

The differential cardiotoxicity profiles observed among various TKI classes can be partially attributed to their unique target specificities and off-target effects. In the context of EGFR-mutated NSCLC, a comprehensive network meta-analysis of 89 RCTs encompassing 29,813 participants revealed that both first-generation (odds ratio [OR] 1.51) and third-generation EGFR-TKIs (OR 2.18) are associated with a significantly increased risk of cardiac adverse events compared to placebo. Notably, within the third-generation inhibitors, osimertinib exhibited the highest risk (OR 2.53) (Ma et al., 2025). Regarding Bruton tyrosine kinase (BTK) inhibitors, a Bayesian network meta-analysis indicated that acalabrutinib (risk ratio [RR] 0.43) and zanubrutinib (RR 0.34) are associated with significantly reduced risks of atrial fibrillation in comparison to ibrutinib (Kritya et al., 2025). Beyond cardiovascular toxicity, TKI resistance continues to pose a significant clinical challenge, particularly in chronic myeloid leukemia (CML). Emerging strategies to address resistance include the exploration of phytoconstituents that target kinase resistance pathways through molecular docking and dynamics simulations (Kashyap et al., 2025). These findings collectively underscore that, notwithstanding the efficacy advantages of TKIs, the challenges of on-target resistance and off-target cardiovascular toxicity represent substantial barriers to sustained therapeutic success. This highlights the critical need for a thorough risk-benefit evaluation in clinical decision-making.

In terms of safety, the results of this meta-analysis demonstrate that TKI use is associated with a significantly higher risk of cardiovascular toxicity compared with placebo. Subgroup analysis based on drug targets showed notable variation in MACE risk across different TKI classes. Among the four target categories, EGFR-TKIs exhibited the highest MACE risk. Subgroup analyses based on drug targets indicated that VEGFR-TKIs and PDGFR-TKIs did not exhibit a significant association with the three-point MACE composite. This observation is biologically plausible, as these pathways are predominantly implicated in hypertension, which is explicitly excluded from the FDA/EMA-endorsed MACE definition. The lack of a significant association for these targets with the standardized MACE endpoint, despite their well-documented hypertensive effects, supports the discriminant validity of employing this definition in the current context. Furthermore, it highlights the necessity of analyzing hypertension as a separate safety outcome, rather than conflating it with the MACE composite. Beyond the components encompassed within the MACE definition, we incorporated additional cardiovascular adverse events (CVAEs) to facilitate a more extensive and comprehensive evaluation of the cardiovascular toxicity profile associated with TKIs. Among the four target categories, EGFR-TKIs exhibited the highest CVAE risk, followed by VEGFR-TKIs, PDGFR-TKIs, and C-kit-TKIs. Significant heterogeneity was observed among studies reporting CVAEs, with the Haas E2805 trial identified as the major source of heterogeneity (Haas et al., 2015). This randomized, double-blind trial assigned patients to nine 6-week cycles of sunitinib 50 mg daily (28 days on, 14 days off), sorafenib 400 mg twice daily, or placebo. The findings showed that continued exposure to sunitinib or sorafenib led to a moderate increase in CVAEs, consistent with other RCTs. However, the E2805 trial reported a lower incidence of significant left ventricular ejection fraction (LVEF) decline during the first 6 months and demonstrated that dose reduction did not mitigate CVAE risk. These discrepancies are likely attributable to the study’s reliance on LVEF as the sole measure of cardiac toxicity, a limitation that warrants further investigation in future research. Further subgroup analysis comparing single-target and multi-target TKIs revealed that single-target TKIs were associated with a higher risk of CVAEs. Because the single-target agents included in this study primarily targeted EGFR, this result aligns with the earlier finding that EGFR-TKIs carry the greatest cardiovascular risk.

The observation that single-target EGFR-TKIs confer higher cardiotoxicity than multi-target TKIs is counterintuitive because multi-target agents are generally associated with broader off-target adverse effects. Several hypotheses may explain this paradox. First, EGFR is not only expressed in tumor cells but also plays essential physiological roles in cardiomyocytes and vascular endothelium. Activation of EGFR signaling promotes cell survival, maintains mitochondrial function, and preserves contractile integrity under stress conditions. Inhibition of EGFR by EGFR-TKIs disrupts these protective pathways, leading to reduced nitric oxide bioavailability, increased oxidative stress, and impaired calcium handling in cardiac myocytes. These molecular disturbances manifest clinically as QT interval prolongation, decreased left ventricular ejection fraction, and heightened susceptibility to arrhythmias (Li et al., 2024). Moreover, EGFR inhibition compromises endothelial barrier function and promotes vascular inflammation, which may accelerate atherosclerosis and contribute to myocardial ischemia and stroke. Many other kinase targets play lesser roles in normal cardiac homeostasis. Thus, selective EGFR inhibition directly disrupts a critical survival pathway in cardiomyocytes, leading to dose-dependent and sometimes irreversible cardiotoxicity. In contrast, multi-target TKIs inhibit several kinases simultaneously, which may activate compensatory mechanisms that partially offset the cardiac effects. Second, underreporting or differential surveillance cannot be ruled out. Multi-target TKI trials often focus on class-specific adverse events, whereas cardiac events such as QT prolongation may be underdetected if routine ECG monitoring is not mandated. Conversely, EGFR-TKIs have a well-recognized risk of QT prolongation, prompting more rigorous cardiac monitoring and thus higher reported event rates. Third, we compared single-target EGFR-TKIs with multi-target TKIs. If other single-target TKIs had been included, the pattern might have differed. Therefore, our finding should be interpreted as specific to EGFR-TKIs versus multikinase inhibitors rather than as a general rule for all single-target agents.

We also performed separate analyses for different categories of CVAEs. Arrhythmias exhibited the highest risk with EGFR-TKIs, followed by VEGFR-TKIs. Additionally, the incidence of QT prolongation was higher with EGFR-TKIs than with VEGFR-TKIs or PDGFR-TKIs. Because QT prolongation is a form of arrhythmia, these findings support the internal consistency of the subgroup analyses. Previous studies have similarly reported that, compared with conventional therapies, EGFR-TKIs and VEGFR-TKIs are associated with elevated risks of QT prolongation, heart failure, and other CVAEs (Chaar et al., 2018; Ghatalia et al., 2015; Abdel-Qadir et al., 2017; Khokhar et al., 2024). No significant difference in the incidence of heart failure was found between the experimental and control groups. This may be explained by differences in baseline patient characteristics or the relatively small sample sizes in some trials. The meta-analysis also revealed that hypertension risk was highest with PDGFR-TKIs, followed by VEGFR-TKIs. This is likely attributable to the vascular-targeted mechanisms of these agents, which make patients more susceptible to developing hypertension (Manzat Saplacan et al., 2017; Andrae et al., 2008; Kang et al., 2023).

To explore whether the risk of CVAEs associated with TKIs varies across different malignancies, we conducted an exploratory subgroup analysis comparing trials enrolling predominantly patients with NSCLC with those including other solid tumors. The increased risk of TKI-associated CVAEs was consistently observed in both NSCLC studies and the pooled “other solid tumors” category. These findings are consistent with the results of our primary analysis without tumor-type stratification, further supporting the robustness of the association between TKIs and increased CVAE risk. Furthermore, in our subgroup analysis stratified by both tumor type and TKI target class, EGFR-TKIs consistently exhibited higher cardiotoxicity than other TKI classes. This result aligns with our prior analysis without tumor-type stratification, suggesting that the elevated cardiotoxicity of EGFR-TKIs is minimally influenced by tumor type.

Efficacy analysis revealed that TKI-treated patients had significantly longer PFS than placebo-treated patients. Subgroup analysis by drug target further indicated that VEGFR-TKIs provided the greatest improvement in PFS, followed by EGFR-TKIs and PDGFR-TKIs. The point estimate for c-Kit TKIs suggested a numerically smaller benefit, but the pooled HR was not statistically significant; therefore, no firm ranking can be assigned to this class. This ranking differed from the CVAE risk profile, with the primary discrepancy observed for EGFR-TKIs. EGFR-TKIs are well known to be associated with a higher risk of pulmonary toxicity, particularly interstitial lung disease, which represents a major cause of treatment-related mortality in patients receiving EGFR-targeted therapy (Ren et al., 2012; Hosomi et al., 2020; Dong et al., 2024). We hypothesize that the shorter PFS observed for EGFR-TKIs relative to VEGFR-TKIs in our analysis may be partly attributable to this pulmonary toxicity, which could negatively affect clinical outcomes and overall patient survival. Additionally, no significant difference in all-cause mortality (OS) was found between the TKI and placebo groups. This may be explained by several factors. First, many patients likely received subsequent therapies after the trial period, which could have influenced long-term survival outcomes. Second, OS encompasses all causes of death, not only those related to cancer progression, which may dilute or obscure treatment effects in statistical analyses (Pilz et al., 2012).

After assessing the overall risk of CVAEs associated with TKI therapy, this study conducted subgroup analyses based on different TKI targets to determine how specific molecular profiles influence cardiovascular risk. Several meta-analyses have examined the cardiovascular safety of TKIs; however, most have focused on a single TKI or tumor type and have not separately stratified outcomes by kinase target or analyzed individual MACE components (Chen et al., 2024; Hou et al., 2021). In contrast, our target-stratified approach demonstrates that EGFR-TKIs carry a significantly higher risk of QT prolongation and arrhythmias, whereas VEGFR-TKIs are more strongly associated with hypertension. This granularity provides clinically actionable insights and represents an advance over previous aggregate analyses. Taken together, our findings highlight the need for class-specific cardiovascular monitoring when using TKIs, with efficacy data serving as a secondary consideration in risk–benefit decision-making.

## 5 Limitations

This study has several limitations. First, some analyses demonstrated substantial heterogeneity, which may be attributable to differences in treatment protocols, diagnostic criteria for outcomes, and the types and severities of diseases represented in the included studies. Our analysis grouped TKIs according to their primary nominal targets. However, many TKIs exhibit broad kinase inhibition profiles that extend beyond their designated primary target. Consequently, these differences may confound the observed associations between target classes and cardiotoxicity. Therefore, the findings should be interpreted as drug-class associations rather than direct biological evidence that a specific kinase target is solely responsible for the observed cardiovascular events. Second, the safety evaluation focused specifically on QT prolongation, heart failure, hypertension, and arrhythmias. Other important cardiovascular adverse events, such as myocardial infarction or reduced LVEF, were not assessed, and the risks associated with these outcomes remain unclear. Additional research is necessary to address these gaps. Third, the safety and dosing regimens of TKIs are closely related; however, we were unable to analyze the impact of different dosing strategies because equivalent dose conversions across TKIs could not be established, and disease types varied across the included studies. Fourth, because multi-target TKIs act on multiple receptors, each target may influence cardiovascular risk. Our analysis focused on four shared targets, which may not fully account for the potential contributions of additional targets. Fifth, some safety outcomes were based on adverse event reports from study investigators rather than adjudication by blinded cardiology experts, which may have introduced reporting bias. Finally, because baseline cardiovascular risk factors were not analyzed at the individual-patient level, we cannot provide specific guidance on TKI selection according to cardiovascular status. Future studies incorporating baseline risk stratification are needed to determine whether patients with certain cardiac conditions might benefit from avoiding specific TKIs. The choice of TKI should balance oncologic efficacy against the observed class-specific cardiovascular risks, and routine cardiac monitoring should be considered for all patients receiving high-risk agents.

## 6 Conclusion

This study demonstrates that, based on efficacy outcomes, VEGFR-TKIs provide the greatest improvement in PFS, followed by EGFR-TKIs, PDGFR-TKIs, and c-Kit TKIs. In terms of safety, EGFR-TKIs pose the highest risk of cardiotoxicity, followed by VEGFR-TKIs, PDGFR-TKIs, and C-kit-TKIs. Single-target TKIs, particularly EGFR-TKIs, carry a higher risk of MACEs than multi-target TKIs. EGFR-targeted agents are more strongly associated with arrhythmias and QT prolongation, whereas PDGFR-targeted TKIs show a greater association with hypertension. These findings highlight the importance of considering each patient’s cardiovascular profile when selecting a TKI.

## Data Availability

The original contributions presented in the study are included in the article/Supplementary Material, further inquiries can be directed to the corresponding author.
