# Supplementary material for: Target-specific cardiotoxicity of tyrosine kinase inhibitors: a Systematic Review and meta-analysis
Source: Front Pharmacol. 2026 Jul 23;17:1807099. doi: 10.3389/fphar.2026.1807099 (PMC13443044; doi:10.3389/fphar.2026.1807099)
Supplement: Supplementary file 1 [file Supplementaryfile1.docx]

eFigure. 1 Risk-of-bias assessment of the included RCTs using the Cochrane Risk of Bias Tool.

eFigure 2. Summary risk-of-bias assessment of the included RCTs using the Cochrane Risk of Bias Tool.

Risk of bias was assessed using the Cochrane Collaboration’s tool for randomized controlled trials, Color coding: green = low risk; yellow = unclear risk; red = high risk, “+” = low risk of bias; “?” = unclear risk; “-” = high risk. Domains assessed according to Cochrane RoB 1.0 tool: random sequence generation, allocation concealment, blinding of participants/personnel, blinding of outcome assessment, incomplete outcome data, selective reporting, and other bias.

eFigure 3. Venn diagram of overlapping TKI therapeutic targets (EGFR, VEGFR, PDGFR, and C-kit).

The diagram shows the four target families of TKIs: EGFR, VEGFR, PDGFR and c‑Kit. Each closed shape represents one target family. Overlapping areas indicate that some TKIs inhibit multiple kinase families simultaneously. Non‑overlapping regions represent target‑specific inhibitors.

Abbreviations: TKI, tyrosine kinase inhibitor; EGFR, epidermal growth factor receptor; VEGFR, vascular endothelial growth factor receptor; PDGFR, platelet‑derived growth factor receptor, C-kit, KIT proto-oncogene receptor.

eFigure. 4 Forest plot of CVAEs comparing tyrosine kinase inhibitors (TKIs) with placebo in patients with advanced malignancies.

For CVAEs, a random-effects model was used (I^2^ = 52%, *P* < 0.00001). Squares represent the effect estimate (odds ratio [OR]) for each individual study; the size of each square is proportional to the weight assigned to that study in the meta-analysis. Horizontal lines indicate the 95% confidence interval (CI) for each study. The diamond at the bottom of the forest plot represents the pooled effect estimate; the width of the diamond corresponds to the 95% CI of the pooled estimate.

Abbreviations: TKI, tyrosine kinase inhibitor; CI, confidence interval; OR, odds ratio; CVAEs, cardiovascular adverse events.

eFigure. 5 Subgroup analysis of MACE risk by TKI target class (EGFR-TKIs, VEGFR-TKIs, PDGFR-TKIs, and c-Kit TKIs) compared with placebo.

For subgroup analysis, a fixed-effects model was used (EGFR: I^2^ = 10%, *P* < 0.00001; VEGFR: I^2^ = 61%, *P* < 0.0001; PDGFR: I^2^ = 11%, *P* = 0.002; c-Kit: I^2^ = 49%, *P* = 0.02). Squares represent the effect estimate (OR) for each individual study; the size of each square is proportional to the weight assigned to that study in the meta-analysis. Horizontal lines indicate the 95% CI for each study. The diamond at the bottom of each forest plot represents the pooled effect estimate; the width of the diamond corresponds to the 95% CI of the pooled estimate.

Abbreviations: TKI, tyrosine kinase inhibitor; EGFR, epidermal growth factor receptor; VEGFR, vascular endothelial growth factor receptor; PDGFR, platelet-derived growth factor receptor; C-kit, KIT proto-oncogene receptor; CI, confidence interval; OR, odds ratio; MACEs, major adverse cardiovascular events.

eFigure 6. Subgroup analysis of arrhythmia risk by TKI target class compared with placebo.

For subgroup analysis, a fixed‑effects model was used (EGFR I² = 0%, P <0.00001; VEGFR I² = 24%, P <0.00001; PDGFR I² = 0%, P =0.12; C-kit I² = 0%, P =0.96). Squares represent the effect estimate (hazard ratio, HR) of each individual study; the size of each square is proportional to the weight assigned to that study in the meta‑analysis. Horizontal lines indicate the 95% confidence interval (CI) of each study’s HR. The diamond at the bottom of each forest plot represents the pooled effect estimate; the width of the diamond corresponds to the 95% CI of the pooled HR.

eFigure 7. Subgroup analysis of QT interval prolongation risk by TKI target class compared with placebo.

For subgroup analysis, a fixed‑effects model was used (EGFR I² = 9%, P <0.00001; VEGFR/PDGFR I² = 60%, P=0.22). Squares represent the effect estimate (hazard ratio, HR) of each individual study; the size of each square is proportional to the weight assigned to that study in the meta‑analysis. Horizontal lines indicate the 95% confidence interval (CI) of each study’s HR. The diamond at the bottom of each forest plot represents the pooled effect estimate; the width of the diamond corresponds to the 95% CI of the pooled HR.

eFigure 8. Subgroup analysis of hypertension risk by TKI target class compared with placebo.

For subgroup analysis, a fixed‑effects model was used (EGFR I² = 0%, P =0.57; VEGFR I² = 73%, P < 0.00001; PDGFR I² = 77%, P < 0.00001; C-kit I² = 0%, P=0.71). Squares represent the effect estimate (hazard ratio, HR) for each individual study; the size of each square is proportional to the weight assigned to that study in the meta‑analysis. Horizontal lines indicate the 95% confidence interval (CI) of each study’s HR. The diamond at the bottom of each forest plot represents the pooled effect estimate; the width of the diamond corresponds to the 95% CI of the pooled HR.

eFigure 9. Forest plot of heart failure associated with TKIs compared with placebo.

For heart failure, a random‑effects model was used (I² = 0%, P =0.66). Squares represent the effect estimate (hazard ratio, HR) for each individual study; the size of each square is proportional to the weight assigned to that study in the meta‑analysis. Horizontal lines indicate the 95% confidence interval (CI) of each study’s HR. The diamond at the bottom of each forest plot represents the pooled effect estimate; the width of the diamond corresponds to the 95% CI of the pooled HR.

eFigure 10. Forest plot of cardiogenic death associated with TKIs compared with placebo.

For cardiogenic death, a fixed‑effects model was used (I² = 0%, P =0.22). Squares represent the effect estimate (hazard ratio, HR) for each individual study; the size of each square is proportional to the weight assigned to that study in the meta‑analysis. Horizontal lines indicate the 95% confidence interval (CI) of each study’s HR. The diamond at the bottom of each forest plot represents the pooled effect estimate; the width of the diamond corresponds to the 95% CI of the pooled HR.

eFigure 11. Subgroup analysis of cardiogenic death by TKI target class compared with placebo.

For subgroup analysis of cardiogenic death, a fixed‑effects model was used (EGFR I² = 0%, P =0.12; VEGFR I² = 0%, P = 0.65; PDGFR I² = 0%, P = 0.65). Squares represent the effect estimate (hazard ratio, HR) of each individual study; the size of each square is proportional to the weight assigned to that study in the meta‑analysis. Horizontal lines indicate the 95% confidence interval (CI) of each study’s HR. The diamond at the bottom of each forest plot represents the pooled effect estimate; the width of the diamond corresponds to the 95% CI of the pooled HR.

eFigure 12. Forest plot of CVAEs in NSCLC associated with TKIs compared with placebo.

For CVAEs in NSCLC, a random‑effects model was used (I² = 33%, P =0.008). Squares represent the effect estimate (hazard ratio, HR) of each individual study; the size of each square is proportional to the weight assigned to that study in the meta‑analysis. Horizontal lines indicate the 95% confidence interval (CI) of each study’s HR. The diamond at the bottom of each forest plot represents the pooled effect estimate; the width of the diamond corresponds to the 95% CI of the pooled HR.

eFigure 13. Subgroup analysis of CVAEs in NSCLC by TKI target class compared with placebo.

For subgroup analysis, a fixed‑effects model was used (EGFR I² = 0%, P =0.0002; VEGFR I² = 0%, P = 0.009).Squares represent the effect estimate (hazard ratio, HR) of each individual study; the size of each square is proportional to the weight assigned to that study in the meta‑analysis. Horizontal lines indicate the 95% confidence interval (CI) of each study’s HR. The diamond at the bottom of each forest plot represents the pooled effect estimate; the width of the diamond corresponds to the 95% CI of the pooled HR.

eFigure 14. Forest plot of CVAEs in other solid tumors associated with TKIs compared with placebo.

For CVAEs in other solid tumors, a random‑effects model was used (I² = 63%, P < 0.0001).Squares represent the effect estimate (hazard ratio, HR) of each individual study; the size of each square is proportional to the weight assigned to that study in the meta‑analysis. Horizontal lines indicate the 95% confidence interval (CI) of each study’s HR. The diamond at the bottom of each forest plot represents the pooled effect estimate; the width of the diamond corresponds to the 95% CI of the pooled HR.

eFigure 15. Subgroup analysis of CVAEs in other solid tumors by TKI target class compared with placebo.

For subgroup analysis, a fixed‑effects model was used (EGFR I² = 0%, P < 0.0001; VEGFR I² = 66%, P < 0.00001; PDGFR I² = 1%, P = 0.002; C-kit I² = 49%, P = 0.02). Squares represent the effect estimate (hazard ratio, HR) of each individual study; the size of each square is proportional to the weight assigned to that study in the meta‑analysis. Horizontal lines indicate the 95% confidence interval (CI) of each study’s HR. The diamond at the bottom of each forest plot represents the pooled effect estimate; the width of the diamond corresponds to the 95% CI of the pooled HR.

eFigure 16. Forest plot of overall survival (OS) comparing TKIs with placebo in patients with advanced malignancies.

For OS, a fixed‑effects model was used (I² = 16%, P = 0.93). Squares represent the effect estimate (hazard ratio, HR) of each individual study; the size of each square is proportional to the weight assigned to that study in the meta‑analysis. Horizontal lines indicate the 95% confidence interval (CI) of each study’s HR. The diamond at the bottom of each forest plot represents the pooled effect estimate; the width of the diamond corresponds to the 95% CI of the pooled HR.

Abbreviations: OS, overall survival.

eFigure 17. Forest plot of progression-free survival (PFS) comparing TKIs with placebo in patients with advanced malignancies.

For PFS, a random‑effects model was used (I² = 91%, P = 0.0001). Squares represent the effect estimate (hazard ratio, HR) of each individual study; the size of each square is proportional to the weight assigned to that study in the meta‑analysis. Horizontal lines indicate the 95% confidence interval (CI) of each study’s HR. The diamond at the bottom of each forest plot represents the pooled effect estimate; the width of the diamond corresponds to the 95% CI of the pooled HR.

Abbreviations: PFS, Progression Free Survival.

eFigure 18. Subgroup analysis of PFS by TKI target class compared with placebo.

For subgroup analysis, a fixed‑effects model was used (EGFR I² = 42%, P = 0.005; VEGFR I² = 92%, P < 0.00001; PDGFR I² = 90%, P < 0.0001; C-kit I² = 64%, P = 0.13). Squares represent the effect estimate (hazard ratio, HR) of each individual study; the size of each square is proportional to the weight assigned to that study in the meta‑analysis. Horizontal lines indicate the 95% confidence interval (CI) of each study’s HR. The diamond at the bottom of each forest plot represents the pooled effect estimate; the width of the diamond corresponds to the 95% CI of the pooled HR.

eFigure 19. Sensitivity analysis of MACE risk associated with TKIs.

Each horizontal line (or point) represents the pooled odds ratio (OR) and its 95% confidence interval (CI) after excluding the study listed on the left. The vertical dashed line indicates the original pooled OR (or null effect line, if appropriate). The solid diamond (or point) at the bottom shows the original overall estimate including all studies.

eFigure 20. Funnel plot assessing publication bias among studies reporting MACEs associated with TKIs.

Each circle represents an individual study. The vertical dashed line indicates the pooled effect estimate (log odds ratio or log hazard ratio).

eTable 1. Search strategies for PubMed, the Cochrane Library, Embase, Wanfang Data, and CNKI from database inception through June 2026.

| #1 | TKIs [Title/Abstract] |
| --- | --- |
| #2 | EGFR-TKIs[Title/Abstract] |
| #3 | VEGFR-TKIs[Title/Abstract] |
| #4 | Gefetinib[Title/Abstract] |
| #5 | Erlotinib[Title/Abstract] |
| #6 | Lenvatinib[Title/Abstract] |
| #7 | Vandetanib[Title/Abstract] |
| #8 | Cabozantinib[Title/Abstract] |
| #9 | Axitinib[Title/Abstract] |
| #10 | Pazopanib[Title/Abstract] |
| #11 | Sunitinib[Title/Abstract] |
| #12 | Sorafenib[Title/Abstract] |
| #13 | Osimertinib[Title/Abstract] |
| #14 | #1 OR #2 OR #3 OR #4 OR #5 OR #6 OR #7 OR #8 OR #9 OR #10 OR #11 OR #12 |
| #15 | Cardiotoxicity [Title/Abstract] |
| #16 | Cardiac arrest [Title/Abstract] |
| #17 | heart failure [Title/Abstract] |
| #18 | QT prolongationsyndrome [Title/Abstract] |
| #19 | Myocardial ischemia [Title/Abstract] |
| #20 | Hypertension [Title/Abstract] |
| #21 | Arrhythmia [Title/Abstract] |
| #22 | MACE [Title/Abstract] |
| #23 | cardiovascular events [Title/Abstract] |
| #24 | #15 OR #16 OR #17 OR #18 OR #19 OR #20 OR #21 OR #22 OR #23 |
| #25 | "Randomized Controlled Trial" [Publication Type] |
| #26 | random trial[Title/Abstract] |
| #27 | random trails[Title/Abstract] |
| #28 | randomized controlled[Title/Abstract] |
| #29 | clinical trials, randomized[Title/Abstract] |
| #30 | trials, randomized clinical[Title/Abstract] |
| #31 | controlled clinical trials, randomized[Title/Abstract] |
| #32 | random[Title/Abstract] |
| #33 | randomize[Title/Abstract] |
| #34 | RCT[Title/Abstract] |
| #35 | #24 OR #25 OR #26 OR #27 OR #28 OR #29 OR #30 OR #31 OR #32 OR #33 OR #34 |
| #36 | #14 And #24 And #35 |

eTable 2. Primary molecular targets of the included tyrosine kinase inhibitors (TKIs).

| Drug name | Targets | | | | | | | |
| --- | --- | --- | --- | --- | --- | --- | --- | --- |
| Gefitinib | EGFR |  |  |  |  |  |  |  |
| Erlotinib | EGFR |  |  |  |  |  |  |  |
| Lenvatinib |  | VEGFR | FGFR | RET | PDGFR |  |  |  |
| Vandetanib | EGFR | VEGFR |  |  |  |  |  |  |
| Cabozantinib |  | VEGFR |  |  |  | HGFR |  |  |
| Axitinib |  | VEGFR |  |  | PDGFR |  |  |  |
| Pazopanib |  | VEGFR |  |  | PDGFR |  | C-Kit |  |
| Sunitinib |  | VEGFR |  |  | PDGFR |  |  |  |
| Sorafenib |  | VEGFR |  |  | PDGFR |  | C-Kit | FLT3 |
| Osimertinib | EGFR |  |  |  |  |  |  |  |

eTable 3. CVAEs reported in the prescribing information (drug labeling) of the included TKIs.

| Drug name | CVAEs listed in drug labeling |
| --- | --- |
| Gefitinib | QT prolongationsyndrome |
| Erlotinib | Arrhythmia, Myocardial infarction, Myocardial ischemia |
| Lenvatinib | QT prolongationsyndrome, Heart failure, LVEF |
| Vandetanib | QT prolongationsyndrome, TDP |
| Cabozantinib | Cardiac arrest |
| Axitinib | Heart failure |
| Pazopanib | QT prolongationsyndrome, TDP |
| Sunitinib | QT prolongationsyndrome, LVEF |
| Sorafenib | QT prolongationsyndrome, Myocardial infarction, Myocardial ischemia, Heart failure |
| Osimertinib | QT prolongationsyndrome, LVEF, Heart failure |
